# A pragmatic randomised controlled trial of tailored pulmonary rehabilitation in participants with difficult-to-control asthma and elevated body mass index

**DOI:** 10.1186/s12890-022-02152-2

**Published:** 2022-09-24

**Authors:** Helen Clare Ricketts, Varun Sharma, Femke Steffensen, Anna Goodfellow, Elaine Mackay, Gordon MacDonald, Duncan S. Buchan, Rekha Chaudhuri, Douglas C. Cowan

**Affiliations:** 1 College of Medical, Veterinary and Life Sciences, University of Glasgow, Glasgow, UK; 2 Glasgow Clinical Research Facility, Glasgow Royal Infirmary, Glasgow, UK; 3 Pulmonary Rehabilitation Team, Glasgow Royal Infirmary, Glasgow, UK; 4 Respiratory Department, 4th Floor, Walton Building, Glasgow Royal Infirmary, 84 Castle Street, Glasgow, G4 0SD UK; 5 Division of Sports and Exercise, University of the West of Scotland, Lanarkshire, UK; 6 Institute of Infection, Inflammation and Immunity, University of Glasgow, Glasgow, UK

**Keywords:** Asthma, Difficult-to-control asthma, Pulmonary rehabilitation, Obesity

## Abstract

**Background:**

Difficult-to-control asthma associated with elevated body mass index (BMI) is challenging with limited treatment options. The effects of pulmonary rehabilitation (PR) in this population are uncertain.

**Methods:**

This is a randomised controlled trial of an eight-week asthma-tailored PR programme versus usual care (UC) in participants with difficult-to-control asthma and BMI ≥ 25 kg/m^2^. PR comprised two hours of education and supervised exercise per week, with encouragement for two individual exercise sessions. Primary outcome was difference in change in Asthma Quality of Life Questionnaire (AQLQ) in PR versus UC groups between visits. Secondary outcomes included difference in change in Asthma Control Questionnaire-6 (ACQ6), and a responder analysis comparing proportion reaching minimum clinically important difference for AQLQ and ACQ6.

**Results:**

95 participants were randomised 1:1 to PR or UC. Median age was 54 years, 60% were female and median BMI was 33.8 kg/m^2^. Mean  (SD) AQLQ was 3.9 (+/-1.2) and median (IQR) ACQ6 2.8(1.8–3.6). 77 participants attended a second visit and had results analysed. Median (IQR) change in AQLQ was not significantly different: 0.3 (− 0.2 to 0.6) in PR and − 0.1 (− 0.5 to 0.4) in UC, *p* = 0.139. Mean change in ACQ6 was significantly different: − 0.4 (95% CI − 0.6 to − 0.2) in PR and 0 (− 0.3 to + 0.3) in UC, *p* = 0.015, but below minimum clinically important difference. In ACQ6 responder analysis, minimum clinically important difference was reached by 18 PR participants (54.5%) versus 10 UC (22.7%), *p* = 0.009. Dropout rate was 31% between visits in PR group, and time to completion was significantly prolonged in PR group at 94 (70–107) days versus 63 (56–73) in UC, *p* < 0.001.

**Conclusions:**

PR improved asthma control and reduced perceived breathlessness in participants with difficult-to-control asthma and elevated BMI. However, this format appears to be suboptimal for this population with high drop-out rates and prolonged time to completion.

*Trial registration* Clinicaltrials.gov. ID NCT03630432. Retrospectively registered, submitted May 26th 2017, posted August 14th 2018.

## Background

Difficult-to-control asthma is a term which suggests asthma with ongoing symptoms or frequent exacerbations, despite significant treatment. Significant treatment describes either high dose inhaled corticosteroids (ICS) plus long-acting β2 agonist (LABA) or leukotriene receptor antagonist (LTRA); or medium dose ICS plus LABA/LTRA and one other drug, or frequent/continuous oral corticosteroids (OCS) [1]. Evidence indicates obesity can both lead to and worsen asthma [2]. Obese asthma is associated with increased symptoms [3], frequent exacerbations [4, 5] and resistance to traditional therapies including ICS [6, 7]. In an analysis of 2225 patients registered with British Thoracic Society (BTS) Difficult Asthma Registry, mean BMI was 30.8 kg/m^2^ (SD 7.1) [8]. Obesity rates are increasing worldwide, with an almost threefold increase since 1975 [9]. Experts recommend personalisation of asthma treatment with identification of treatable traits [10, 11]. Obese asthma is a phenotype that could be specifically targeted.

Pulmonary rehabilitation (PR) describes an exercise and education programme that has proven beneficial in respiratory conditions including chronic obstructive pulmonary disease (COPD) [12]. Benefits in this population include improvements in quality of life [13] and mental health [14]. The role of PR in asthma is unclear. A Cochrane review of physical training in asthma suggested it was safe and led to improvements in cardiopulmonary fitness, but with no improvements in lung function [15]. BTS and Scottish Intercollegiate Guidelines Network (SIGN) asthma guidelines recommend exercise should be advised as part of general lifestyle advice to everyone with asthma. If overweight, then weight loss advice is recommended [1]. Few studies have evaluated the effects of PR in asthma. A recent small study (n = 34) demonstrated weight reduction and improved asthma control after intensive PR [16]. Another feasibility study suggested some improvements but acknowledged high dropout rates [17].

## Methods

### Study aim and design

Our objective was to evaluate the impact of a tailored PR programme in overweight/obese individuals with difficult-to-control asthma. We aimed to assess effects on asthma-related quality of life and control, as well as other measures of disease burden, exercise tolerance, activity levels and mental health.

This was an unblinded, randomised controlled parallel group trial of asthma tailored PR in individuals with difficult-to-control asthma who were overweight/obese. Participants were randomised 1:1 to PR or usual care (UC). Randomisation was by a third-party drawing from an envelope. Study visits took place at baseline (V1) and eight weeks, or completion of eight PR sessions (V2). The study took place between May 2017 and December 2020 in Glasgow Royal Infirmary. It was registered with clinicaltrials.gov (ID NCT03630432) and approved by West of Scotland Regional Ethics Committee (reference 16/WS/0200).

### Study participants

Participants were recruited from tertiary asthma clinics across the Greater Glasgow region. Participants were aged 18–80 years, with BMI ≥ 25 kg/m^2^. Asthma was diagnosed according to Global Initiative for Asthma guidelines [18], with characteristic symptoms and at least one of: 12% and 200mls increase in forced expiratory volume in 1 s (FEV_1_) after inhaled/nebulised short-acting β-2 agonist (SABA), or ≥ 4 weeks of anti-inflammatory treatment, or between visits; or positive bronchial challenge test (PC_20_ methacholine or histamine < 8 mg/ml or PD_15_ mannitol < 635 mg). Asthma was uncontrolled despite at least high dose ICS and LABA [19], with either ≥ 2 courses OCS, ≥ 1 asthma-related hospitalisation, or asthma control questionnaire-6 (ACQ6) score > 1.5 within the previous year. Exclusion criteria included an exacerbation requiring OCS and/or antibiotics within four weeks; significant co-morbidity; mobility problems likely to influence study conduct; pregnancy/breastfeeding; and intensive care unit (ICU) admission or commencement of biologic therapy within six months.

A substantial amendment was approved in August 2018. This removed FEV_1_/FVC ratio ≤ 70% and Medical Research Council (MRC) Dyspnoea Score ≤ 3 from inclusion criteria. Within exclusion criteria, minimum time from ICU admission to recruitment was reduced to 6 months from 12, and a 6 month period following discontinuation of antifungal, biologic therapy or Airsonett device was removed. These changes were made to widen recruitment and were not expected to impact on study outcomes.

Individuals expressing an interest in participation received a Patient Information Sheet and were invited to provide written informed consent prior to commencing study.

### PR programme

The PR course lasted eight weeks, with one in-hospital session per week comprising an hour each of education and exercise. International guidelines recommend at least two supervised weekly sessions [12, 20], but acknowledging attendance may be an issue, we pragmatically reduced to one supervised session and encouraged two further independent sessions each week. Compliance with this was not monitored.

The educational component was delivered on a rolling basis by multidisciplinary staff. Topics covered are listed in Table 1. The exercise was delivered in a hospital gym by the PR Team. Asthma stability was verbally confirmed before starting each session, and pre-exercise administration of SABA inhaler was encouraged. Exercises were taken from the local PR programme and comprised a warm-up followed by resistance and aerobic exercises. Training intensity was individually tailored based on distance walked during baseline six-minute walk test (6MWT) and current activity profile as assessed on verbal interview by the physiotherapists. There was progressive increase in repetitions/resistance each week.

**Table 1:** Pulmonary rehabilitation educational topics

Educational topics
What is asthma: diagnosis, co-morbidities
Asthma treatments
Treatment, inhaler technique and personalised asthma management
Breathing control and chest clearance
Health promotion including healthy eating
Asthma, general health and physical activity
Asthma, mental health and well-being
Benefits of exercise, anxiety management and relaxation

#### Exercises

Most participants began with one set of 12 repetitions of each strength exercise in the first week. This was then increased to two sets of 12 and then three sets of 12 repetitions as the weeks progressed, depending on how well the participant had managed the previous week. A description of strength exercises and progression follows:

- Leg extensions—involve sitting in a chair and raising the leg from floor to horizontal. This was progressed with the addition of ankle weights (1–3 kg)
- Bicep curls—progressed with the addition of dumbbells (0.5–5 kg)
- Sit-to-stand—rising from sitting in a chair to standing up, no progression in weights
- Step ups—stepping from floor onto a box approximately 30 cm off the ground, progressed by addition of ankle weights, and increased box height
- Pole raises—raising a plastic pole from waist height to shoulders then above head to full arm extension, progressed by the addition of weights (1–6 kg)
- Knee lifts—standing on the spot then lifting knee until thigh perpendicular with the floor, progressed by the addition of ankle weights

Aerobic exercises involved:

- Walking—walking on the flat around the room at a comfortable pace for 3 min. This was advanced by walking for a longer time period, at a brisker pace, then up and down a ramp.
- Exercise bike—pedalling on a stationary exercise bike with low resistance for 3 min. This was advanced by increasing both resistance and time

Some participants already exercised regularly and managed longer distances on the baseline 6MWT. They had the exercises adapted to make them more challenging. Some participants were advised to spend a longer time on aerobic exercises. Some participants had more difficult strength training exercises using weights machines and heavier weights. The exercise for each participant was tailored to their ability at baseline and progressed throughout the eight sessions.

If sessions were missed participants were contacted by telephone or email, and reattendance was encouraged. All participants were asked to attend eight sessions. At completion, participants were encouraged to continue regular exercise by referral to community-based facilities.

### Study measurements

At V1, information including demographics, medical history and medications was obtained by participant interview and using electronic medical records. Participants completed several questionnaires including asthma quality of life questionnaire (AQLQ) [21, 22]; ACQ6 [23, 24]; MRC dyspnoea score [25]; and hospital anxiety and depression scale (HADS) [26].

Height and weight were recorded, and BMI calculated. Participants performed fraction of exhaled nitric oxide (FeNO) using NIOX VERO machine (Circassia Pharmaceuticals, Morrisville, USA). Peak expiratory respiratory flow (PEFR) and spirometry were performed before and 15 min after inhaled salbutamol, on a Vitalograph (Maids Moreton, U.K.) spirometer. Blood samples were taken for eosinophil count. Two 6MWTs were carried out with furthest distance and corresponding Borg score at completion used for analysis.

Each participant wore an ActiGraph wGTX3-BT (ActiGraph, Pensacola, Florida, USA) accelerometer on their non-dominant wrist continually for seven days (except when bathing/swimming) to estimate physical activity (PA).

At completion of V1, participants were randomised, with PR course starting one week later. Both groups were advised to continue pre-study asthma management, with changes allowed as clinically indicated. Inhaler technique was reviewed and corrected if necessary. All participants were provided with a personalised asthma management plan.

V2 was scheduled for eight weeks after V1. V2 was postponed until eight PR sessions were completed, if necessary. V2 followed the same format. Anyone who attended V2 was regarded as completing PR, no matter how many sessions they attended, hence analysis was intention-to-treat.

Those randomised to the UC group had V2 scheduled for eight weeks later, and no other contact between visits. They were offered the opportunity to complete PR following V2.

Following accelerometer return, data was downloaded using ActiLife v.6.14.3 (ActiGraph, USA). Files were exported into R v3.6.3 (R Foundation for Statistical Computing, Vienna, Austria) and processed using the GGIR package v2.1.0 [27]. This detected non-wear time, abnormally high values and auto-calibrated raw tri-axial signals. It calculated Euclidean Norm Minus One averaged over 5-s epochs in milli-gravitational (m*g*) units [28]. Inactive time was defined as time accumulated below acceleration of 30 m*g*; light PA (LPA) time between 30–99 m*g* [29] and moderate-vigorous PA (MVPA) time ≥ 100 m*g* acceleration. Files were excluded from subsequent analyses if post-calibration error was > 0.01 *g*, there were < 4 days (defined as ≥ 16 h per day) [30], including one weekend day, of valid wear or wear data was not present for each 15-min period of the 24-h cycle.

### Statistical analysis

Baseline characteristics and results are expressed as mean with standard deviation (SD), median and interquartile range (IQR) or numbers and proportions. Analysis was on the basis of intention to treat, with everyone who attended V2 included in analysis, regardless of number of sessions completed. Primary outcome was difference in change in AQLQ between visits for PR versus UC groups.

The minimum clinically important difference (MCID) for AQLQ is 0.5 [24]. Mean (SD) AQLQ for a similar population is 3.5(1.2) (unpublished local data contributed to BTS DAR). To demonstrate a difference of 0.5 mean change between visits, a sample size of 180 was calculated, assuming α 0.05, β 0.2 and power 0.8. It was considered benefits may be larger than anticipated, and was agreed with regional ethics committee at the outset, that an interim analysis would be performed after recruitment of 100. This coincided with the start of the Covid-19 pandemic and no further recruitment was possible due to legal guidelines on face-to-face contact.

Normality testing was performed with D’Agostino-Pearson test. At baseline, comparisons were made using Chi-squared or Fisher’s exact test for proportions, unpaired t test for normally distributed data, and Mann–Whitney *U* test for skewed data.

Data obtained from individuals attending both V1 and V2 were used to compare effects of PR with UC. Change for each individual was calculated; then mean/median change for each group compared using unpaired t or Mann–Whitney *U* test. A responder analysis compared proportion of individuals achieving MCID of 0.5 points improvement in ACQ6 [24] and AQLQ [22] using Chi-squared test. In post-hoc analysis, FeNO and eosinophil levels were compared between ACQ6 and AQLQ responders/non-responders. A *p*-value of < 0.05 was considered statistically significant. Statistical tests were performed using GraphPad Prism v9 (GraphPad Software, San Diego).

## Results

**Fig. 1:**
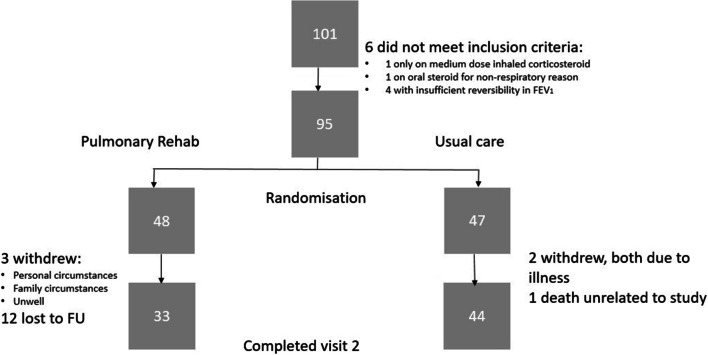
A flowchart demonstrating recruitment, randomisation and follow-up

101 individuals gave informed consent to participate. Six were excluded as inclusion/exclusion criteria were not met, and 95 were randomised; 48 to PR and 47 to UC (Fig. 1).

Baseline characteristics are displayed in Table 2. Median (IQR) age was 54 (47–64) years and 57 (60%) were female. The commonest co-morbidities were gastro-oesophageal reflux disease (80%), allergic rhinitis (72%) and psychological illness (64%). Median number of co-morbidities was 6 (5–7). Participants had a high treatment burden, with 30 (32%) taking regular OCS. 20% received biologics. Median BMI was 33.8 (29.6–38.9) kg/m^2^ with 70 (74%) obese. Baseline ACQ6 was 2.8 (1.8–3.6) and AQLQ 3.9 (1.2). With the exception of montelukast, there were no significant differences between groups at baseline.

**Table 2:** Characteristics at baseline of all participants recruited

	Overall n = 95	Pulmonary rehabilitation group (PR) n = 48	Usual care group (UC) n = 47	*p* value: PR versus UC
Age, years	54 (47–64)	53 (47–61)	56 (47–65)	0.287
Sex				
Female	57 (60)	28 (58.3)	27 (61.7)	0.900
Male	38 (40)	20 (41.7)	18 (38.3)	
Smoking: Ex-smoker	41 (43.2)	19 (39.6)	22 (46.8)	0.7621
Lifelong non-smoker	47 (49.5)	25 (52.1)	22 (46.8)	
Current smoker	7 (7.4)	4 (8.3)	3 (6.4)	
Pack years	20 (10–35)	20 (10–35)	20 (8–34)	0.931
Age at asthma diagnosis	31 (7–47)	33 (9–48)	30 (5–46)	0.455
Duration of asthma, years	21 (10–39)	19 (6–39)	25(14–39)	0.176
Atopy	61 (64.2)	31 (64.6)	30 (63.8)	0.891
Allergic rhinitis	68 (71.6)	35 (72.9)	33 (70.2)	0.949
Perennial rhinitis	46 (48.4)	23 (47.9)	23 (48.9)	0.916
Nasal polyps	14 (14.7)	5 (10.4)	9 (19.1)	0.362
Nasal surgery	19 (20.0)	6 (12.5)	13 (27.7)	0.112
Eczema	20 (21.1)	8 (16.7)	12 (25.5)	0.419
GORD	76 (80.0)	38 (79.2)	38 (80.9)	0.959
DFB/VCD	17 (17.9)	12 (25.0)	5 (10.6)	0.119
Psychological illness	61 (64.2)	32 (66.7)	29 (61.7)	0.771
Emphysema	8 (8.4)	2 (4.2)	6 (12.8)	0.159
Bronchiectasis	14 (14.7)	7 (14.6)	7 (14.9)	0.805
SAFS/ABPA	18 (18.9)	10 (20.8)	8 (17.0)	0.832
Diabetes mellitus	14 (14.7)	5 (10.4)	9 (19.1)	0.362
Hypertension	24 (25.3)	11 (22.9)	13 (27.7)	0.767
Cardiac disease	17 (17.9)	7 (14.6)	10 (21.3)	0.560
Osteopenia/osteoporosis	35 (36.8)	18 (37.5)	17 (36.2)	0.938
SABA nebs	35 (36.8)	19 (39.6)	16 (34.0)	0.729
LAMA	78 (82.1)	41 (85.4)	37 (78.7)	0.560
ICS/LABA	95 (100)	48 (100)	47 (100.0)	> 0.999
BDP equivalent dose, mcg	2000 (1600–2000)	2000 (1600–2400)	1600 (1600–2000)	0.106
Prednisolone maintenance	30(31.6)	16(33.3)	14(29.8)	0.880
Prednisolone dose, mg	6.3 (5.6–6.9)	10.0 (5.0–10.0)	5.0 (5.0–7.5)	0.232
Montelukast	66 (69.5)	39 (81.3)	27 (57.4)	0.022
Theophylline	38 (40.0)	21 (43.8)	17 (36.2)	0.586
Azithromycin	13 (13.7)	6 (12.5)	7 (14.9)	0.967
Omalizumab	11 (11.6)	3 (6.3)	8 (17.0)	0.187
Mepolizumab	8 (8.4)	3 (6.3)	5 (10.6)	0.486
Antihistamine	61 (64.2)	30 (62.5)	31 (66.0)	0.891
Nasal steroid	42 (44.2)	24 (50.0)	18 (38.3)	0.346
PPI/H2A	72 (75.8)	37 (77.1)	35 (74.5)	0.954
Exacerbations in last year	4.0 (2.0–5.0)	3.5 (2.0–5.3)	4.0 (2.0–5.0)	0.990
GP attendances in last year	2 (0–5)	3 (0–5)	2 (1–4)	0.771
A&E attendances in last year	0 (0–1)	0 (0–1)	0 (0–1)	0.829
Hospital admissions in last year	0 (0–1)	0 (0–0)	0 (0–0)	0.328
ICU admissions in last year	0 (0–0)	0 (0–0)	0 (0–0)	0.745
BMI, kg/m^2^	33.8 (29.6–38.9)	33.8 (29.6–37.8)	33.1 (29.6–40.6)	0.916
MRC dyspnoea scale	3 (2–4)	3 (2–4)	3 (2–4)	0.423
ACQ6	2.8 (1.8–3.6)	2.8 (1.5–3.8)	2.8 (2.2–3.5)	0.448
AQLQ overall	3.9 ± 1.2	4.1 ± 1.3	3.7 ± 1.0	0.132
AQLQ symptom domain	3.9 ± 1.3	4.2 ± 1.5	3.7 ± 1.1	0.114
AQLQ activity domain	3.8 ± 1.2	3.9 ± 1.3	3.7 ± 1.6	0.452
AQLQ emotional domain	4.0 ± 1.6	4.3 ± 1.6	3.8 ± 1.5	0.134
AQLQ environmental domain	4.1 ± 1.5	4.3 ± 1.5	3.8 ± 1.5	0.075
HADS anxiety score	9.0 ± 4.8	8.5 ± 4.7	9.4 ± 4.9	0.377
HADS depression score	8.1 ± 4.3	8.1 ± 4.3	8.2 ± 4.3	0.904
Eosinophils (× 10^9^/L)	0.3 (0.1–0.4)	0.3 (0.2–0.5)	0.2 (0.1–0.4)	0.160
FeNO (ppb)	24 (14–49)	21 (13–48)	24 (16–50)	0.531
PEFR (L/min)	398.2 ± 102.6	409.0 ± 104.8	387.2 ± 99.1	0.305
pre-BD FEV_1_ (% predicted)	71.9 ± 16.8	73.0 ± 16.4	70.7 ± 17.1	0.518
pre-BD FEV_1_/FVC %	65 (59–71)	66 (62–72)	65 (58–70)	0.296
% change FEV_1_post-BD	4.8 (− 0.9 to 12.2)	4.7 (− 2.2 to 13.4)	4.8 (2.6–11.1)	0.787
6MWT, metres	390 (315–450)	410 (349–450)	390 (263–428)	0.162
Borg score post-6MWT	2.0 (1.0–3.0)	2.5 (1.0–3.0)	2.0 (1.0–3.0)	0.783
Accelerometry- inactive time (minutes per day)	1170 (1107–1237)	1177 (1114–1238)	1150 (1104–1239)	0.515
Accelerometry- time in LPA (minutes per day)	218 (169–267)	211 (164–250)	236 (170–288)	0.229
Accelerometry- time spent in MVPA (minutes per day)	48 (28–72)	51 (32–74)	40 (27–68)	0.260

Values expressed as number (proportion), mean ± SD or median (IQR) unless otherwise specified

*PR* pulmonary rehabilitation, *UC* usual care control group, *GORD* gastro-oesophageal reflux disease, *DFB* dysfunctional breathing, *VCD* vocal cord dysfunction, *SAFS* severe asthma with fungal sensitisation, *ABPA* allergic bronchopulmonary aspergillosis, *SABA* short acting beta-2 agonist, *LABA* long acting beta-2 agonist, *LAMA* long acting muscarinic antagonist, *ICS* inhaled corticosteroid, *BDP* beclometasone diproprionate dose equivalent, *PPI* proton pump inhibitor, *H2A* H2 receptor antagonist, *A and E* accident and emergency department, *GP* General Practitioner, *ICU* intensive care unit, *BMI* body mass index, *MRC* Medical Research Council dyspnoea score, *ACQ6* Asthma Control Questionnaire 6, *AQLQ* Asthma Quality of Life Questionnaire, *HADS* Hospital Anxiety and Depression Scale, *FeNO* fraction of exhaled nitric oxide, *PEFR* peak expiratory flow rate, *pre-BD* pre-bronchodilator, *FEV1* forced expiratory volume in 1s, *FVC* forced vital capacity, *post-BD* post bronchodilator, *6MWT* 6 min walk test, *LPA* light physical activity, *MVPA* moderate to vigorous physical activity

77 participants attended V2 and were included in analysis, 33 (69%) in PR group and 44 (94%) in UC. Within PR group, 28 (85%) completed eight PR sessions, five ≤ 5 sessions, and mean (SD) sessions attended was 7.1 (2.3). Intended time between visits was 56 days, but median was 94 (70–107) days in PR and 63 (56–73) in UC, *p* < 0.001. This was due to non-attendance at PR sessions prolonging time to completion.

### Primary outcome

**Fig. 2:**
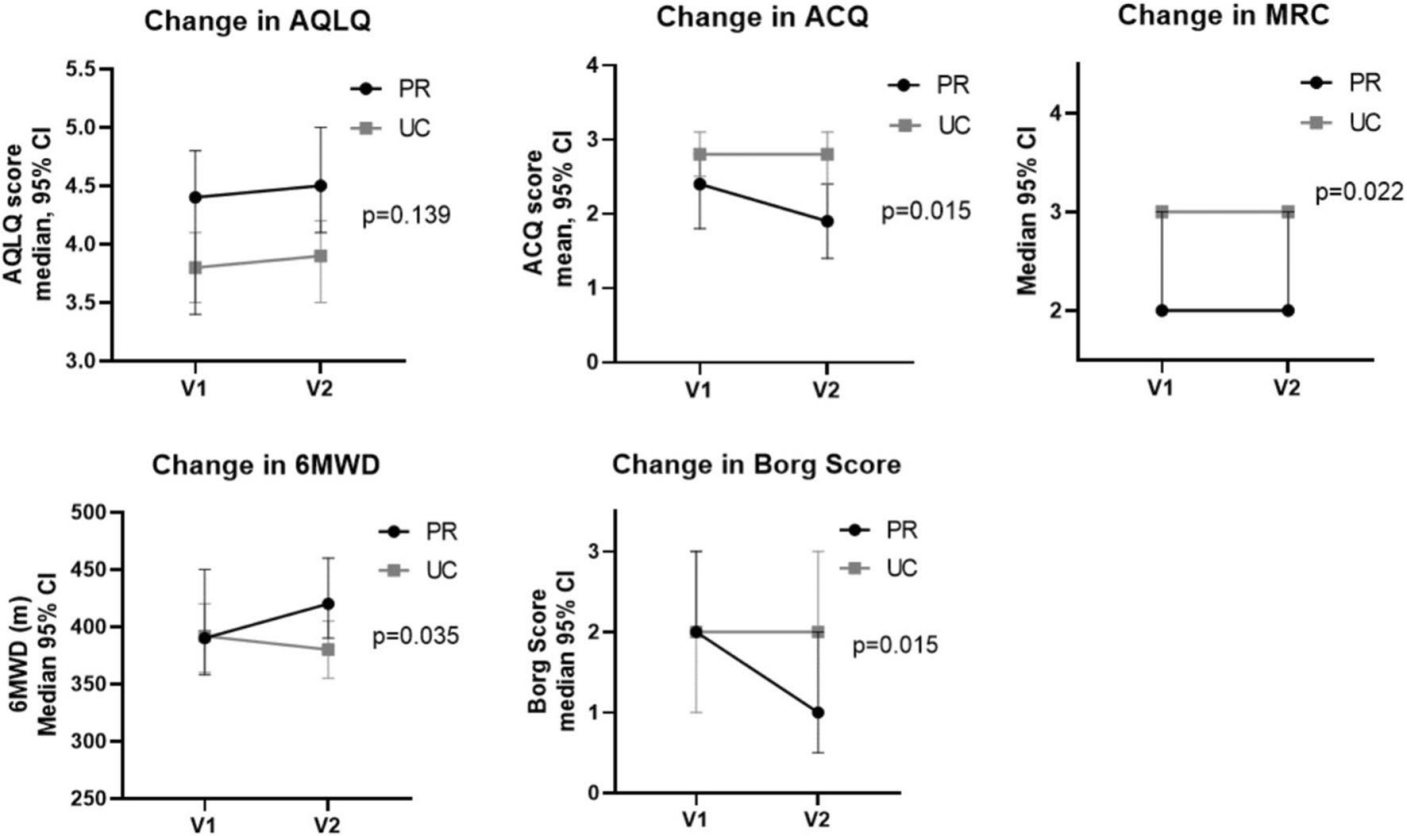
Graphical representation of key results. *AQLQ* asthma quality of life questionnaire, *PR* pulmonary rehabilitation group, *UC* usual care group, *V1* visit 1, *V2* visit 2, *CI* confidence intervals, *ACQ6* 6 point version asthma control questionnaire, *MRC* Medical Research Council Dyspnoea Scale score, *6MWD* six minute walk distance

Results are displayed in Table 3/Fig. 2. Mean (SD) AQLQ at V1 was 4.4 (1.2) in PR group and 3.8 (1.0) in UC, *p* = 0.037. At V2, it was 4.5 (1.2) in PR and 3.9 (1.1) in UC, *p* = 0.018. Median (IQR) change was not significantly different: 0.3 (− 0.2 to 0.6) in PR and − 0.1 (− 0.5 to 0.4) in UC, *p* = 0.139. As significant differences were observed between groups at V1 and V2, post-hoc multiple regression analysis adjusting for baseline was performed. This confirmed no significant difference in change between groups.

**Table 3:** Key results of completers

		PR group (n = 33)	UC group (n = 44)	*p*-value PR versus UC
Overall AQLQ	V1	4.4 ± 1.2	3.8 ± 1.0	**0.037**
	V2	4.5 ± 1.2	3.9 ± 1.1	**0.018**
	Change	0.3 (− 0.2 to 0.6)	− 0.1 (− 0.5 to 0.4)	0.139
AQLQ symptom	V1	4.4 ± 1.5	3.8 ± 1.1	0.062
	V2	4.6 ± 1.4	3.9 ± 1.2	**0.022**
	Change	0.4 (− 0.3 to 0.7)	0.0 (− 0.6 to 0.5)	0.179
AQLQ activity	V1	4.1 ± 1.3	3.8 ± 1.1	0.221
	V2	4.4 ± 1.2	3.8 ± 1.1	**0.045**
	Change	0.5 (− 0.4 to 1.0)	− 0.1 (− 0.6 to 0.5)	0.057
AQLQ Emotional	V1	4.6 ± 1.5	3.9 ± 1.5	**0.036**
	V2	5.0 (3.6–6.2)	4.0 (2.9–5.0)	**0.013**
	Change	0.2 (− 0.2 to 0.6)	0.0 (− 0.75 to 0.75)	0.248
AQLQ environmental	V1	4.8 ± 1.3	3.9 ± 1.5	**0.007**
	V2	4.5 ± 1.5	4.0 ± 1.6	0.186
	Change	− 0.2 (− 0.8 to 0.5)	0.0 (− 0.5 to 0.7)	0.320
ACQ6	V1	2.3 ± 1.4	2.8 ± 1.0	0.103
	V2	1.9 ± 1.4	2.8 ± 1.2	**0.018**
	Change*	− 0.4 (− 0.6 to − 0.2)	0.0 (− 0.3 to 0.3)	**0.015**
MRC dyspnoea score	V1	2 (2–4)	3 (2–4)	0.414
	V2	2 (2–3)	3 (2–4)	**0.080**
	Change	0 (− 1 to 0)	0 (0–1)	**0.022**
HADS anxiety	V1	8 ± 5	9 ± 5	0.269
	V2	8 ± 5	9 ± 5	0.104
	Change	− 1 ± 3	0 ± 3	0.332
HADS depression	V1	9 (4–10)	8 (5–12)	0.723
	V2	8 (4–11)	8 (4–11)	0.296
	Change	− 1 (− 3 to 1)	0 (− 2 to 1)	0.361
BMI kg/m^2^	V1	33.8 (29.8–38.0)	33.0 (29.3–40.1)	0.804
	V2	34.1 (29.8–38.3)	33.1 (29.5–40.6)	0.933
	Change	− 0.1 (− 0.7 to 0.7)	0.1 (− 0.2 to 0.6)	0.209
Eosinophils (× 10^9/L)	V1	0.30 (0.20–0.50)	0.20 (0.10–0.40)	0.096
	V2	0.20 (0.10–0.43)	0.25 (0.10–0.40)	0.994
	Change	0.00 (− 0.10 to 0.00)	0.00 (− 0.10 to 0.10)	0.057
FeNO(ppb)	V1	32 (13–53)	24 (15–49)	0.919
	V2	22 (13–68)	24 (12–41)	0.628
	Change	− 4 (− 11 to 4)	− 4 (− 13 to 3)	0.563
Pre-BD FEV_1_/FVC ratio	V1	65 ± 9	64 ± 9	0.523
	V2	66 ± 11	66 ± 11	0.900
	Change	1 ± 5	2 ± 6	0.194
Pre-BD FEV_1_% predicted	V1	77 (65–85)	71 (61–83)	0.406
	V2	74 (64–89)	74 (61–89)	0.754
	Change	3 (− 6 to 8)	2 (− 3 to 6)	0.982
% change in FEV_1_ post BD	V1	− 0.65 (− 3.09 to 9.18)	4.7 (2.5–11.65)	0.097
	V2	2.48 (− 0.51 to 7.69)	4.07 (− 0.99 to 7.79)	0.960
	Change	2.75 (− 4.72 to 7.67)	− 1.71 (− 7.60 to 4.15)	0.170
6MWD (metres)	V1	390 (345–458)	392 (278–439)	0.618
	V2	420 (368–468)	380 (301–430)	0.055
	Change	20 (− 5 to 40)	− 10 (− 40 to 25)	**0.035**
Borg score	V1	2 (1–3)	2 (0.63–3)	0.597
	V2	1 (0–2)	2 (1–3)	**0.009**
	Change	− 1 (− 2 to 0)	0 (− 1 to 1)	**0.015**
Accelerometry: Inactive time (min d^−1^)	V1	1177 (1114–1238)	1150 (1104–1239)	0.515
	V2	1175 (1093–1234)	1175 (1096–1241)	0.841
	Change	11 (− 53 to 32)	− 4 (− 35 to 84)	0.274
Accelerometry: LPA (min d^−1^)	V1	211 (164–250)	236 (170–288)	0.253
	V2	236 (170–288)	228 (170–290)	0.425
	Change	− 8 (− 18 to 34)	− 4 (− 61 to 27)	0.296
Accelerometry: MVPA (min d^−1^)	V1	51 (32–74)	40 (27–68)	0.260
	V2	44.7 (30.1–80.3)	38.9 (24.9–63.3)	0.319
	Change	− 1 (− 9 to 15)	0 (− 11 to 9)	0.361

Significant values are highlighted in bold

Values are expressed as mean ± SD, median (IQR) or *mean (95% CI)

*PR* pulmonary rehabilitation, *UC* usual care, *AQLQ* asthma quality of life questionnaire, *ACQ* asthma control questionnaire, *MRC* Medical Research Council dyspnoea score, *HAD* hospital anxiety and depression scale, *BMI* body mass index, *FeNO* fraction of exhaled nitric oxide, ppb parts per billion, *pre-BD* pre-bronchodilator, *FEV*_*1*_ forced expiratory volume in 1s, *FVC* forced vital capacity, *6MWD* six minute walk distance, *LPA* light physical activity, *MVPA* moderate-to-vigorous physical activity

There were no significant differences in change in AQLQ domains, although there was a trend towards benefit within activity domain; + 0.5 (− 0.4 to 1) in PR and − 0.1 (− 0.6 to 0.5) in UC, *p* = 0.057.

### Secondary outcomes

**Fig. 3:**
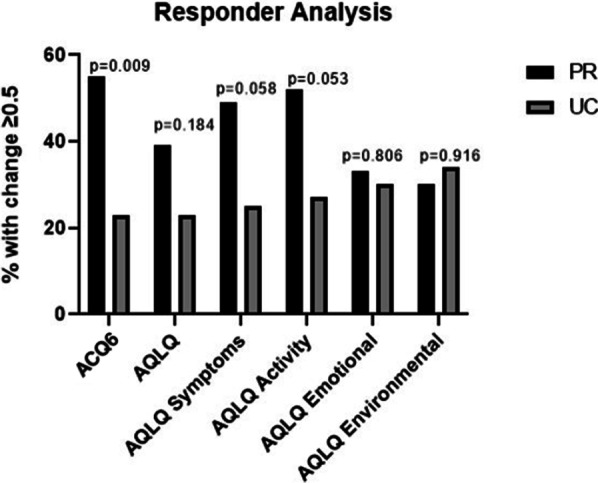
Bar chart showing those who met the minimum clinically important difference for each questionnaire. *ACQ6* 6 point version asthma control questionnaire, *AQLQ* asthma quality of lie questionnaire, *PR* pulmonary rehabilitation group, *UC* usual care group

There was no difference in proportion of participants reaching MCID for improvement in overall AQLQ: 13 (39%) in PR and 10 (23%) in UC, *p* = 0.184 (Table 4/Fig. 3). There were trends towards differences in symptom (*p* = 0.058) and activity domains (*p* = 0.053).

**Table 4:** Participants meeting minimum clinically important difference

	PR group (n = 33)	UC group (n = 44)	*p* value
Δ overall AQLQ ≥ + 0.5	13 (39)	10 (23)	0.184
Δ AQLQ symptoms ≥ + 0.5	16 (49)	11 (25)	0.058
Δ AQLQ activity ≥ + 0.5	17 (52)	12 (27)	0.053
Δ AQLQ emotional ≥ + 0.5	11 (33)	13 (30)	0.806
Δ AQLQ environmental ≥ + 0.5	10 (30)	15 (34)	0.916
Δ ACQ6 ≥ − 0.5	18 (55)	10 (23)	0.009

Values expressed as number and %

*AQLQ* asthma quality of life questionnaire, *ACQ6* 6-point asthma control questionnaire

Mean (SD) ACQ6 at V1 was 2.3 (1.4) in PR and 2.8 (1.0) in UC, *p* = 0.103. At V2 it was 1.9 (1.4) in PR and 2.8 (1.2) in UC, *p* = 0.018. Mean change in ACQ6 was − 0.4 (95% CI − 0.6 to − 0.2) in PR group versus 0 (− 0.3 to + 0.3) in UC, *p* = 0.015 (Table 3/Fig. 2). There was a significant difference in proportion of participants reaching MCID for ACQ6: 18 (55%) in PR versus 10 (23%) in UC, *p* = 0.009 (Table 4/Fig. 3). In addition, the proportion with clinically significant worsening (≥ + 0.5) was higher in the UC group: 15 (34%) compared with 2(6%) in PR, *p* = 0.008.

MRC dyspnoea score at V1 was median (IQR) 2 (2–4) in PR and 3 (2–4) in UC, *p* = 0.414, and at V2 was 2 (2–3) in PR and 3 (2–4) in UC, *p* = 0.008. Median change was significantly different: 0 (− 1 to 0) in PR versus 0 (0–1) in UC, *p* = 0.022.

V1 6MWD was median 390 (345–458) metres in PR and 392 (278–439) in UC, *p* = 0.618, and at V2 was 420 (368–468)m in PR and 380 (301–430) in UC, *p* = 0.055. There was a significant difference in change: + 20 (− 5 to + 40) in PR and − 10 (− 40 to + 25) in UC, *p* = 0.035. In addition, median change in Borg breathlessness scale after 6MWT was significantly different: − 1 (− 2 to 0) in PR and no change (− 1 to + 1) in UC, *p* = 0.015.

There were no significant changes in either HADS domain, nor in BMI, eosinophils, FeNO nor spirometry. Accelerometry results at both time points were available for 25 participants in PR and 32 in UC. There were no significant differences in physical activity parameter in accelerometry results between visits.

### Post-hoc analysis

Within PR group, baseline FeNO was significantly lower in ACQ6 responders than non-responders: median (IQR) 18 (8.5–41) and 47 (17–71) respectively, *p* = 0.020; and in AQLQ responders compared to non-responders: median 14 (8.5–44.5) and 40 (19–71), *p* = 0.038 (Table 5/Fig. 3). There were no corresponding significant differences in blood eosinophils.

**Table 5:** Responder analysis- comparing those who met or did not meet the MCID for ACQ6 and AQLQ

	ACQ6 responder, n = 17	ACQ6 non-responder, n = 15	*p* value
Blood eosinophils, mean (95% CI)	0.27 (0.18–0.37)	0.42 (0.26–0.58)	0.095
FeNO, median (IQR)	18 (8.5–41)	47 (17–71)	**0.020**

	AQLQ responder, n = 12	AQLQ non-responder, n = 20	*p* value
Blood eosinophils, mean (95% CI)	0.29 (0.19–0.39)	0.38 (0.24–0.51)	0.294
FeNO, median (IQR)	14 (8.5–44.5)	40 (19–71)	**0.038**

Significant values are highlighted in bold

Blood eosinophils number × 10^9^/L, FENO parts per billion

*ACQ6* asthma control questionnaire (6-point version), *AQLQ* asthma quality of life questionnaire, *FeNO* fraction of exhaled nitric oxide

### Withdrawn patients

The participants who withdrew or were lost to follow up had slightly poorer asthma control at baseline, with mean ACQ6 2.2 (SD 1.4) for completers compared with 3.3 (1.1) for those who dropped out, *p* < 0.011. In addition, AQLQ scores were better at baseline for completers: mean 4.4 (SD 1.2) versus 3.4 (1.2), *p* = 0.008. This may have impacted on whether to attend or to withdraw from the study.

There was only one episode of bronchospasm requiring nebulised SABA during exercise sessions. One participant in UC group died following a cardiac event during the observation period. This was considered unrelated to study.

## Discussion

Difficult-to-control asthma associated with obesity is challenging with limited treatment options. PR is a standard treatment for many chronic lung diseases but its role in asthma remains unclear. In this pragmatic, randomised controlled trial we aimed to evaluate the effects of an asthma-specific PR programme for participants with difficult-to-control asthma and elevated BMI. Although primary outcome was not reached, we found significant improvements in asthma control, symptoms and exercise tolerance which suggest PR may be beneficial in this group. Furthermore, the programme was safe and well-tolerated. However, there were significant numbers of non-completers and delays to completion, suggesting this current format of PR is suboptimal for this group.

The PR programme was delivered by a multidisciplinary team including doctors, nurses and physiotherapists, with input from dietetics. Educational topics aimed to improve understanding of asthma and benefits of physical activity. Informal feedback suggested education and peer support were invaluable. The exercises were adapted from local PR programme and individually tailored based on ability. There was encouragement to complete two further exercise sessions independently, but compliance was not monitored, as such we only have data regarding attendance at one session per week.

In a retrospective cohort study, Turk et al. looked at groups of obese (n = 53) and non-obese (n = 85) asthmatics undergoing 12 weeks of PR comprising 3 h per week of supervised exercise and 4 h of education [31]. 6MWD rose by median (IQR) 50 m (15–84) in non-obese and 45 m in obese group (13–77), *p* < 0.001. Improvement in ACQ was statistically but not clinically significant: − 0.3 points in non-obese, *p* = 0.021 and − 0.4 in obese, *p* = 0.019. These results are similar to ours. A further small study by the same group [32] suggested improvements in ACQ, AQLQ, 6MWD and BMI following 12 weeks PR in obese asthmatics awaiting bariatric surgery.

A recent randomised controlled trial of 34 participants [16] evaluated effects of 12 weeks PR including thrice-weekly high-intensity interval training, 1500 kilocalorie diet and psychological intervention, with or without an online self-management tool, compared to control group who were advised to lose weight and exercise. Both intervention groups had reductions in BMI, but not controls. ACQ improved by − 0.67 (− 1.42 to 0) in PR and − 0.66 (− 1.17 to − 0.33) in PR plus online tool, both *p* < 0.05. Our study involved shorter, less intensive PR, but similar findings.

Although primary outcome was not met, there were trends towards differences for overall AQLQ, plus AQLQ activity and symptom domains in favour of PR. The trial was stopped early after the interim analysis due to the Covid-19 pandemic, meaning power was not reached. It is difficult to predict what outcomes might have been had recruitment continued. The most notable impact was on ACQ6, which improved significantly in PR group with mean reduction of 0.4, just short of MCID of 0.5 [23]. Furthermore, responder analysis for ACQ6 demonstrated 54.5% in PR group reached MCID compared to 22.7% in UC, *p* = 0.009. In addition, the proportion with clinically significant worsening of ACQ6 (≥ + 0.5) was higher in UC, 15 (34.1%), versus 2 (6.1%) in PR, *p* = 0.008.

We demonstrated significant effects of PR on 6MWD, albeit 20 m improvement in PR group being under the 35 m MCID [33]. This is smaller than the improvements seen in COPD PR trials. Reasons for this could include the population being younger and more active at baseline. There were no significant changes in physical activity measured by accelerometry, suggesting this format of programme did not stimulate significant alterations to exercise behaviours.

Our study population had difficult-to-control asthma with many co-morbidities, significant treatment burden, frequent exacerbations and poor AQLQ/ACQ6 scores. This profile associated with T2-high characteristics would allow consideration of biologic treatment, but options in T2-low asthma are limited. Of 95 participants randomised, 17 expressed T2-low features (both eosinophil count < 150/µL and FeNO < 25 ppb [34, 35]), eight in PR group and nine in UC. A post-hoc analysis showed FeNO was significantly lower in responders than non-responders, but with no difference in eosinophil count. This suggests responders may be more likely to display T2-low features [36]. PR could therefore be specifically targeted at obese asthmatics of T2-low endotype, although this would require confirmation.

### Limitations and future directions

This study was underpowered, as the Covid-19 pandemic began immediately after the interim analysis rendering further recruitment impossible. The pandemic impacted other aspects of this study with discontinuation of PR sessions. Face-to-face visits were replaced with telephone calls resulting in some missing data.

Dropout rate was high, 18 between visits. 48 were randomised to PR: 33 attended V2, 3 withdrew and 12 were lost to follow up, which equates to 31% dropping out before completion of PR. This is similar to real-world experience, where approximately 30% commencing PR fail to complete [37, 38]. Time to completion was also prolonged, with median 94 (70–107) days between visits for PR group compared to 63 (56–73) for UC, which may have influenced outcomes. Both drop-out rate and prolonged time to completion were impacted by many of our participants being of working age. Several struggled to attend sessions due to work. Additionally, childcare was an issue for several participants. Indeed, many who met the entry criteria and were approached with information about the study declined to participate for both work and childcare reasons. Asthma exacerbations was another reason for prolonged time to completion in PR group, with 31 participants (40%) having one or more courses of OCS between visits; 15 (48%) of those in PR group and 16 (34%) in UC. It was also noted that participants who withdrew had higher baseline ACQ6 score and lower AQLQ scores, which is likely to reflect poorer asthma control and higher impact of asthma symptoms on ability to exercise and may contribute to the reasons for study withdrawal.

The drop-out rate and prolonged time to completion indicate that the traditional PR format is not ideal for this population of working age adults. Possibilities for improving accessibility, and hopefully attendance and completion, include virtual sessions, community rather than hospital-based classes, and evening sessions.

Other referenced studies [16, 31, 32] involved intensive PR with multiple supervised weekly sessions. We aimed to be pragmatic, therefore included only one supervised session with encouragement for two further independent sessions. We did not record adherence to the additional sessions, and anticipate that many participants did not complete these. It is possible our results were consequently less impressive. It is worth noting that reducing the number of sessions did not improve completion rates.

In addition, exercises were adapted from COPD PR, typically an older, frailer population. Some participants found they were not particularly challenged, which may have resulted in less perceived improvement. Education was delivered on a rolling basis, so if classes were missed some educational talks were too.

Further research is needed to explore the effects of PR in T2-low obese asthma, and clarify optimal programme format. Interactive, live online sessions at a variety of times including evenings and on demand recorded sessions are likely to be more appealing and may improve attendance and completion rates. In addition, this may allow monitoring of number of weekly sessions, and would provide an accessible means of having three sessions per week. Further work could also assess whether delivery of PR in conjunction with dietary intervention adds benefit in obese asthmatics.

## Conclusions

This trial of pulmonary rehabilitation in participants with difficult-to-control asthma and elevated BMI demonstrated statistically significant improvements in asthma control questionnaire score, exercise tolerance (as measured by six minute walk distance), and perception of breathlessness (as demonstrated by Borg score at completion of 6MWT and MRC dyspnoea scale) but effects were small and of uncertain clinical significance. The intervention was safe and well-tolerated. However, this format of face-to-face daytime sessions was not optimal for our participants as demonstrated by the high drop-out rate and prolonged time to completion. Further studies are required to identify the optimal mode of delivery of pulmonary rehabilitation in this population and whether it is associated with clinically relevant benefits.

## Data Availability

The full trial protocol, datasets used and analysed during current study are available from the corresponding author on reasonable request.

## Abbreviations

6MWD: Six-minute walk distance
6MWT: Six-minute walk test
A and E: Accident and emergency department
ABPA: Allergic bronchopulmonary aspergillosis
ACQ6: 6-Point asthma control questionnaire
AQLQ: Asthma-related quality of life questionnaire
BD: Bronchodilator
BDP: Beclometasone diproprionate dose equivalent
BMI: Body mass index
BTS: British Thoracic Society
COPD: Chronic obstructive pulmonary disease
DAR: Difficult Asthma Registry
DFB: Dysfunctional breathing
FEV_1_: Forced expiratory volume in 1s
FeNO: Fraction of exhaled nitric oxide
FVC: Forced vital capacity
GP: General practitioner
GORD: Gastro-oesophageal reflux disease
HADS: Hospital anxiety and depression scale
H2A: H2 receptor antagonist
ICS: Inhaled corticosteroid
ICU: Intensive care unit
LABA: Long-acting beta-2 agonist
LPA: Light physical activity
LTRA: Leukotriene receptor antagonist
MCID: Minimum clinically important difference
MRC: Medical Research Council
MVPA: Moderate to vigorous physical activity
OCS: Oral corticosteroid
PA: Physical activity
PEFR: Peak expiratory respiratory flow rate
PPI: Proton pump inhibitor
PR: Pulmonary rehabilitation
SABA: Short-acting beta-2 agonist
SAFS: Severe asthma with fungal sensitisation
SIGN: Scottish intercollegiate guidelines network
T2: T helper cells type 2
UC: Usual care
V1: Visit 1
V2: Visit 2
VCD: Vocal cord dysfunction
